# Contrasting trends of PM_2.5_ and surface-ozone concentrations in China from 2013 to 2017

**DOI:** 10.1093/nsr/nwaa032

**Published:** 2020-02-28

**Authors:** Yonghong Wang, Wenkang Gao, Shuai Wang, Tao Song, Zhengyu Gong, Dongsheng Ji, Lili Wang, Zirui Liu, Guiqian Tang, Yanfeng Huo, Shili Tian, Jiayun Li, Mingge Li, Yuan Yang, Biwu Chu, Tuukka Petäjä, Veli-Matti Kerminen, Hong He, Jiming Hao, Markku Kulmala, Yuesi Wang, Yuanhang Zhang

**Affiliations:** State Key Laboratory of Atmospheric Boundary Layer Physics and Atmospheric Chemistry (LAPC), Institute of Atmospheric Physics, Chinese Academy of Sciences, Beijing 100029, China; Institute for Atmospheric and Earth System Research, University of Helsinki, Helsinki 00014, Finland; State Key Laboratory of Atmospheric Boundary Layer Physics and Atmospheric Chemistry (LAPC), Institute of Atmospheric Physics, Chinese Academy of Sciences, Beijing 100029, China; China National Environmental Monitoring Center (CNEMC), Beijing 100012, China; State Key Laboratory of Atmospheric Boundary Layer Physics and Atmospheric Chemistry (LAPC), Institute of Atmospheric Physics, Chinese Academy of Sciences, Beijing 100029, China; China National Environmental Monitoring Center (CNEMC), Beijing 100012, China; State Key Laboratory of Atmospheric Boundary Layer Physics and Atmospheric Chemistry (LAPC), Institute of Atmospheric Physics, Chinese Academy of Sciences, Beijing 100029, China; State Key Laboratory of Atmospheric Boundary Layer Physics and Atmospheric Chemistry (LAPC), Institute of Atmospheric Physics, Chinese Academy of Sciences, Beijing 100029, China; Institute for Atmospheric and Earth System Research, University of Helsinki, Helsinki 00014, Finland; State Key Laboratory of Atmospheric Boundary Layer Physics and Atmospheric Chemistry (LAPC), Institute of Atmospheric Physics, Chinese Academy of Sciences, Beijing 100029, China; State Key Laboratory of Atmospheric Boundary Layer Physics and Atmospheric Chemistry (LAPC), Institute of Atmospheric Physics, Chinese Academy of Sciences, Beijing 100029, China; Anhui Institute of Meteorological Sciences, Hefei 230031, China; State Key Laboratory of Atmospheric Boundary Layer Physics and Atmospheric Chemistry (LAPC), Institute of Atmospheric Physics, Chinese Academy of Sciences, Beijing 100029, China; State Key Laboratory of Atmospheric Boundary Layer Physics and Atmospheric Chemistry (LAPC), Institute of Atmospheric Physics, Chinese Academy of Sciences, Beijing 100029, China; State Key Laboratory of Atmospheric Boundary Layer Physics and Atmospheric Chemistry (LAPC), Institute of Atmospheric Physics, Chinese Academy of Sciences, Beijing 100029, China; State Key Laboratory of Atmospheric Boundary Layer Physics and Atmospheric Chemistry (LAPC), Institute of Atmospheric Physics, Chinese Academy of Sciences, Beijing 100029, China; Institute for Atmospheric and Earth System Research, University of Helsinki, Helsinki 00014, Finland; State Key Joint Laboratory of Environment Simulation and Pollution Control, Research Center for Eco-Environmental Sciences, Chinese Academy of Sciences, Beijing 100085, China; Center for Excellence in Regional Atmospheric Environment, Institute of Urban Environment, Chinese Academy of Sciences, Xiamen 361021, China; Institute for Atmospheric and Earth System Research, University of Helsinki, Helsinki 00014, Finland; Institute for Atmospheric and Earth System Research, University of Helsinki, Helsinki 00014, Finland; State Key Joint Laboratory of Environment Simulation and Pollution Control, Research Center for Eco-Environmental Sciences, Chinese Academy of Sciences, Beijing 100085, China; Center for Excellence in Regional Atmospheric Environment, Institute of Urban Environment, Chinese Academy of Sciences, Xiamen 361021, China; State Key Joint Laboratory of Environment Simulation and Pollution Control, School of Environment, Tsinghua University, Beijing 100084, China; Institute for Atmospheric and Earth System Research, University of Helsinki, Helsinki 00014, Finland; State Key Laboratory of Atmospheric Boundary Layer Physics and Atmospheric Chemistry (LAPC), Institute of Atmospheric Physics, Chinese Academy of Sciences, Beijing 100029, China; Center for Excellence in Regional Atmospheric Environment, Institute of Urban Environment, Chinese Academy of Sciences, Xiamen 361021, China; State Key Joint Laboratory of Environmental Simulation and Pollution Control, College of Environmental Sciences and Engineering, Peking University, Beijing 100871, China

**Keywords:** air pollution, clean-air action, particulate matter, surface ozone, China, chemical composition

## Abstract

Although much attention has been paid to investigating and controlling air pollution in China, the trends of air-pollutant concentrations on a national scale have remained unclear. Here, we quantitatively investigated the variation of air pollutants in China using long-term comprehensive data sets from 2013 to 2017, during which Chinese government made major efforts to reduce anthropogenic emission in polluted regions. Our results show a significant decreasing trend in the PM_2.5_ concentration in heavily polluted regions of eastern China, with an annual decrease of ∼7% compared with measurements in 2013. The measured decreased concentrations of SO_2_, NO_2_ and CO (a proxy for anthropogenic volatile organic compounds) could explain a large fraction of the decreased PM_2.5_ concentrations in different regions. As a consequence, the heavily polluted days decreased significantly in corresponding regions. Concentrations of organic aerosol, nitrate, sulfate, ammonium and chloride measured in urban Beijing revealed a remarkable reduction from 2013 to 2017, connecting the decreases in aerosol precursors with corresponding chemical components closely. However, surface-ozone concentrations showed increasing trends in most urban stations from 2013 to 2017, which indicates stronger photochemical pollution. The boundary-layer height in capital cities of eastern China showed no significant trends over the Beijing–Tianjin–Hebei, Yangtze River Delta and Pearl River Delta regions from 2013 to 2017, which confirmed the reduction in anthropogenic emissions. Our results demonstrated that the Chinese government was successful in the reduction of particulate matter in urban areas from 2013 to 2017, although the ozone concentration has increased significantly, suggesting a more complex mechanism of improving Chinese air quality in the future.

## INTRODUCTION

Particulate matter (PM) and ozone are the main pollutants that play important roles in climate change and human health [1–6]. In addition, high concentrations of surface ozone in photochemical pollution have been reported, which weaken net primary production [7–10]. In particular, air-pollution episodes have occurred frequently in the latest decades in China. For example, a series of intensive haze-pollution episodes occurred in eastern China during January of 2013, in which the peak hourly averaged mass concentration of PM_2.5_ exceeded 500 μg m^−3^ in Beijing and its surroundings [11,12]. High concentrations of aerosol precursors (e.g. volatile organic compounds (VOCs), NO_x_, SO_2_ and NH_3_) and secondary aerosol formation, combined with aerosol and boundary-layer feedback, are considered to be responsible for particle pollution and photochemical pollution [13–16]. A recent review clearly summarized that severe haze formation was a synergetic effect of interactions between anthropogenic emissions and atmospheric processes, highlighting that further knowledge about emission sources, physical/chemical mechanisms and interactions with meteorology during haze periods was needed to reveal the causes, mechanisms and trends of haze [17].

From the beginning of 2013, the central government of China took lots of measures to improve the air quality in the Beijing–Tianjin–Hebei (BTH), the Yangtze River Delta (YRD) and the Pearl River Delta (PRD) regions. In particular, the state council announced clean-air action in September of 2013, aiming to reduce concentrations of PM_2.5_ in BTH, YRD and PRD in the next 5 years by as much as 25%, 20% and 15%, respectively. As a response, the local governments began to take practical action to reduce the primary emissions of both gases and PM. For instance, more strict emission standards for thermal power plants, industry and on-road vehicles were promulgated from 2013 [18]. Moreover, the Ministry of Ecology and Environment of China (MEE) established a monitoring network in order to measure the spatio-temporal variation in air pollutants. Invited by the MEE and the state council of China, comprehensive evaluation of the variation in air pollutants from 2013 to 2017 was carried out during the year 2018.

In this study, we show the characteristics of the results based on observed data sets from 2013 to 2017. The results will benefit our knowledge about the current air-pollution situation and policymaking for future air-pollution control.

## RESULTS

### Decreasing trends of PM_2.5_ concentration but increasing ozone-mixing ratio

**Figure 1.**
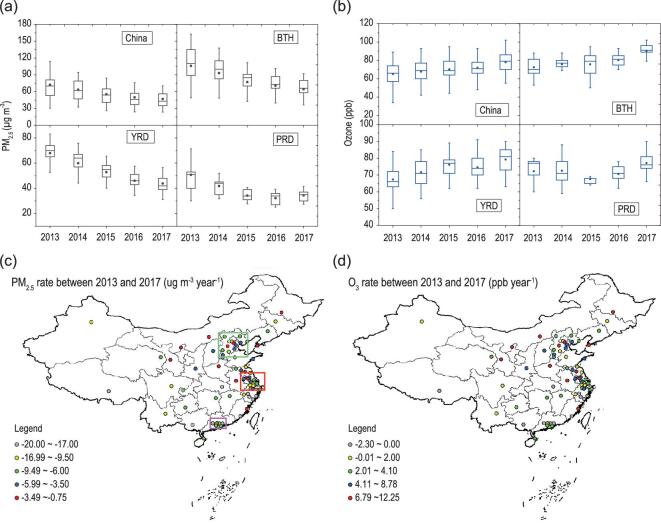
(a) Annual mass concentration of PM_2.5_ in China, Jing-Jin-Ji (BTH), Yangtze River Delta (YRD) and Pearl River Delta (PRD) from 2013 to 2017. The mass concentrations of PM_2.5_ were calculated from 74 key cities in China. The upper and lower boundaries of the boxes represent the 75th and 25th percentiles; the line within the box represents the median value; the whiskers above and below the boxes represent the 90th and 10th percentiles; the points within the box represent the mean value. (b) Annual mixing ratio of ozone in China, BTH, YRD and PRD from 2013 to 2017. (c) Variation ratio of PM_2.5_ concentration in 2017 compared with that in 2013. The BTH region, YRD region and PRD region are marked by green, red and purple squares, respectively. (d) Variation ratio of ozone concentration in 2017 compared with that in 2013.

Figure 1a shows the annual PM_2.5_ concentrations in China and in the BTH, YRD and PRD regions. The annual-average concentration of PM_2.5_ in China, calculated from measurements in 74 cities, was 72.3 ± 37.4 μg m^−3^ in 2013 and the annual-average concentrations in BTH, PRD and YRD were 106.1 ± 36.7, 67.5 ± 13.2 and 47.2 ± 6.6 μg m^−3^, respectively. These values are 1.3–3.0 times the threshold value of 35 μg m^−3^ suggested by the World Health Organization, which clearly demonstrate a serious particle-matter-pollution problem. However, with the implementation of clean-air action, the annual PM_2.5_ concentration in China decreased significantly from 72.3 ± 37.4 to 47.4 ± 20.6 μg m^−3^, the most significant decrease being observed in the BTH region, where the annual-average PM_2.5_ concentration decreased by about 40% from 106.1 to 64.3 μg m^−3^. Among the three regions in eastern China, the PRD region has the lowest PM_2.5_ concentration and, accordingly, showed also the lowest percentage of PM_2.5_ reduction due to the respective small capability of emission reduction. Figure 1c shows the absolute decrease in concentrations from 2013 to 2017 in different cities in China. The most significant reduction of PM_2.5_ concentration also in the absolute sense occurred in the BTH region, with the decrease rate being around 4–20 μg m^−3^ per year. The maximum 8-hour-average 90-percentile (M8A90) ozone-mixing ratio is suggested by the MEE of China to characterize the statistic potential damage of ozone. Figure 1b shows that the annual-average M8A90 in China, BTH, YRD and PRD was equal to 64.3 ± 13.2, 70.4 ± 10.6, 62.4 ± 9.6 and 71.2 ± 9.8 ppb, respectively, in 2013 and that this quantity increased to 77.4 ± 12.6, 91.5 ± 8.7, 76.2 ± 8.4 and 77.3 ± 6.5 ppb in 2017, respectively. As shown in Fig. 1d, the most significant increase in M8A90 occurred in BTH and YRD, the rate increase being in the range of 3–12 ppb per year. Spatially, it seems that the increases in ozone concentrations coincided with the decreases in PM_2.5_ concentrations. Compared with the global distribution of surface ozone, Lu *et al.* [7] have demonstrated that the 4MDA8 (the fourth-highest daily maximum 8-hour average) and Perc98 (98th percentile of hourly concentrations) ozone concentrations were 86.0 ± 14.7 and 80.7 ± 14.1 ppb in China during 2013–2017, which are 20–25% higher than the average values in Europe and the USA.

### Decreasing aerosol-precursor gas concentrations based on field measurements

**Figure 2.**
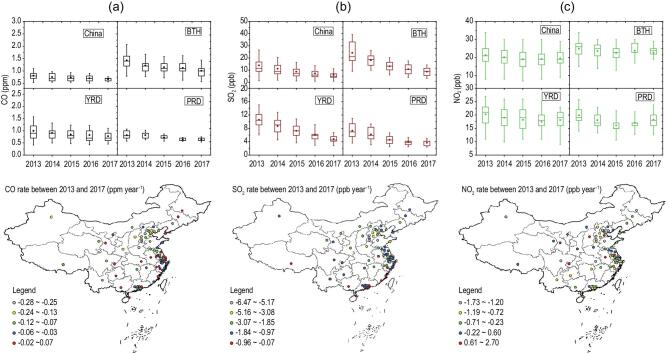
Annual mixing ratio and variation of CO (a), SO_2_ (b) and NO_2_ (c) in China, BTH, YRD and PRD from 2013 to 2017. The mixing ratios were calculated from 74 key cities in China. The upper and lower boundaries of the boxes represent the 75th and 25th percentiles; the line within the box represents the median value; the whiskers above and below boxes represent the 90th and 10th percentiles; the points within the box represent the mean value. The differences between the mixing ratios at 74 cities in 2017 were compared with those in 2013.

Differently from the concurrently decreasing PM_2.5_ and surface ozone in developed countries, such as the USA, significant increases in surface-ozone concentrations took place along with reductions in PM_2.5_ concentrations in corresponding regions in China (e.g. BTH) [19]. Since simultaneous VOC measurements on a national scale are lacking, it is difficult to estimate trends in VOC concentrations over the period 2013–2017. However, considering the similar sources of anthropogenic VOCs and CO, the variability in the CO concentration can be used as a proxy for the variability in anthropogenic VOC concentrations in a specific region [20]. This proxy approach was earlier applied to the analysis of the ozone weekend effect in North China Plain [2]. As shown in Fig. 2a, the CO concentrations in 2013 were equal to 1.0 ± 0.4, 1.4 ± 0.3, 0.8 ± 0.2 and 0.8 ± 0.2 ppm in China, BTH, YRD and PRD, respectively. These concentrations decreased to 0.7 ± 0.3, 1.1 ± 0.3, 0.7 ± 0.1 and 0.7 ± 0.1 ppm in 2017, the corresponding percentages of the decrease being 30%, 21%, 12% and 12%.

The SO_2_ concentrations in 2013 were equal to 14.1 ± 8.2, 24.8 ± 11.2, 10.7 ± 3.0 and 7.2 ± 2.4 ppb in China, BTH, YRD and PRD, respectively, decreasing to 5.5 (12%), 8.0 (13%), 4.1 (13%) and 3.2 ppb (11%) in 2017. As presented in Fig. 2b, the reduction was most significant in BTH, with the rate of decrease at ∼4 ppb per year. SO_2_ emissions come mainly from coal combustion in power plants and residential heating, so the reduction in SO_2_ concentrations should be driven by the decreased emissions of these factors [18,21]. Previous studies have suggested that sulfate is an important component in PM_2.5_, with an average mass fraction of around 18% in urban cities in China [22]. Therefore, the reduction in SO_2_ may explain the reduction in PM_2.5_ concentration to some extent. As a main precursor of nitrate aerosol, the NO_2_ concentrations in 2013 were equal to 21.6 ± 5.2, 25.1 ± 5.0, 21.3 ± 3.8 and 22.2 ± 3.1 ppb in China, BTH, YRD and PRD, respectively (Fig. 2c). These concentrations decreased to 19.2 ± 4.1, 22.5 ± 4.7, 18.2 ± 3.2 and 18.1 ± 2.7 ppb in 2017, respectively, the corresponding percentages of the decreases being equal to 11%, 10%, 15% and 18%. Due to the high number of on-road vehicles and vast emissions of NO_x_ [21], it is still a challenge to reduce NO_2_ and nitrate aerosol concentrations in China. Moreover, the interaction between NO_2_ and SO_2_ could lead to enhanced production of the sulfate aerosol [23,24]; therefore, we suggest that a reduction in NO_x_ emissions is urgent for improving the air quality, especially from industry and vehicles rather than power sectors.

## DISCUSSION

**Figure 3.**
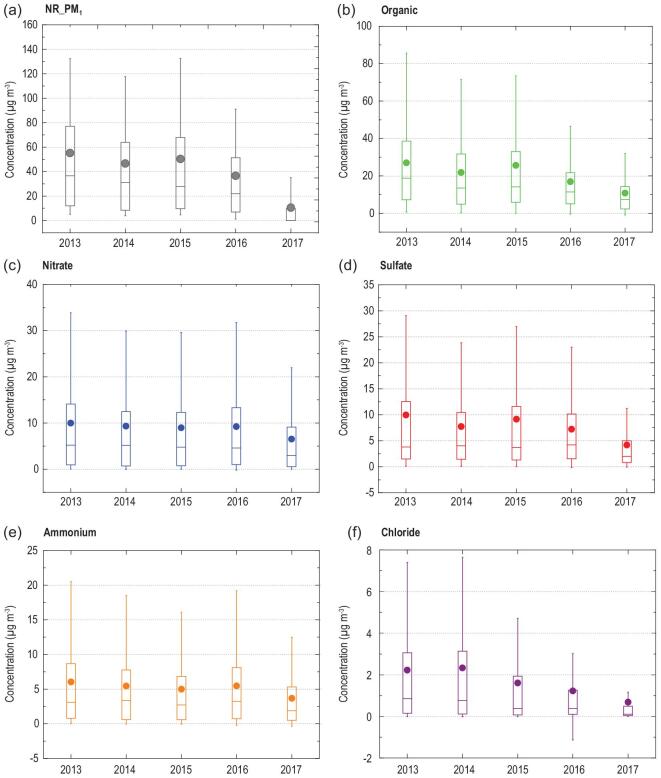
Annual mass concentrations of NR_PM_1_ (a), organic (b), nitrate (c), sulfate (d), ammonium (e) and chloride (f) in urban Beijing by HR-ToF-AMS from 2013 to 2017. The upper and lower boundaries of the boxes represent the 75th and 25th percentiles; the line within the box represents the median value; the whiskers above and below the boxes represent the 90th and 10th percentiles; the points within the box represent the mean value. Note that the annual mass concentrations were calculated from different periods during a year, since AMS was not always working. The period with measurement is shown in the Supplementary Data. The annual-average mass concentrations of organic, nitrate, sulfate, ammonium and chloride were 27.0 ± 26.2, 10.0 ± 12.2, 10.1 ± 14.6, 6.1 ± 7.2 and 2.2 ± 3.1 μg m^−3^, respectively, in 2013 and decreased to 10.8 ± 12.5, 6.5 ± 8.6, 4.2 ± 6.2, 3.7 ± 4.5 and 0.7 ± 1.4 μg m^−3^, respectively, in 2017.

**Figure 4.**
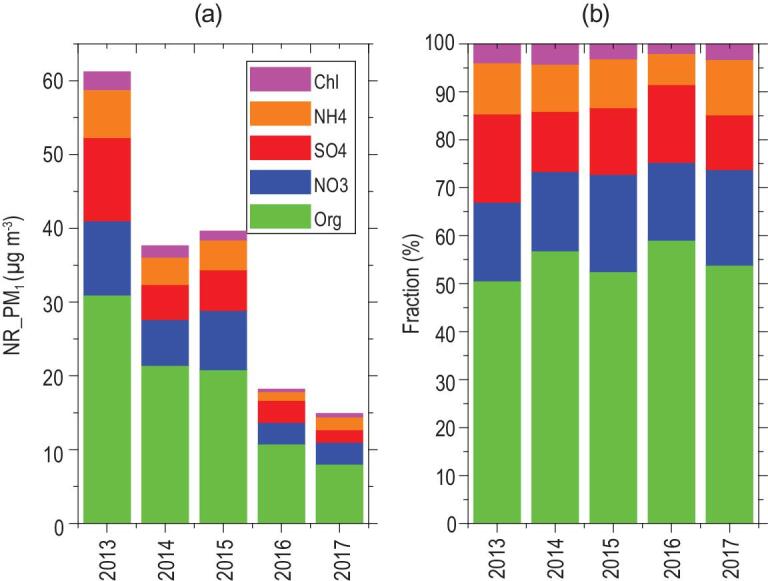
Variation of (a) mass concentration in NR_PM_1_ and (b) mass fraction of organic, nitrate, sulfate, ammonium and chloride during heating seasons from 2013 to 2017.

We suppose that the decreased concentration of PM_2.5_ should be related to decreased aerosol-precursor gas concentrations. VOCs, NO_2_ and SO_2_ are generally considered as precursors of organic,

nitrate and sulfate components in aerosols, respectively [25]. To illustrate how aerosol chemical composition varies with the various aerosol-precursor gas concentration, we take our long-term aerosol composition measurements in urban Beijing as an example. The field measurements showed that the annual-average mass concentrations of organic aerosol, nitrate, sulfate, ammonium and chloride, remarkably decreased between 2013 and 2017 (Fig. 3), consistent with the significant decrease in PM_2.5_ on a regional scale. Considering specifically the heating periods responsible for frequent air-pollution episodes in Beijing during the past decades [26], we can see that the organic aerosol, nitrate, sulfate, ammonium and chloride concentrations in NR_PM_1_ decreased during the heating periods between 2013 and 2017 (Fig. 4). While the mass concentration of these compounds decreased considerably from 2013 to 2017, their mass fractions did not show similarly large changes. The mass fractions of sulfate and chloride decreased from 18.4% to 11.4% and from 3.9% to 3.3%, respectively, whereas the mass fractions of both nitrate and ammonium increased from 16.4% to 20.0% and from 10.7% to 11.6%, respectively.

The formation of surface ozone is determined by VOC and NO_x_ concentration and by the intensity of UV radiation [27]. The increased

surface-ozone concentrations may result from the increased UV radiation, which could photolysis more NO_2_ into NO and consequently increase ozone formation. Hu *et al.* [28] found that the solar-radiation intensity increased by 1.93 W m^−2^ per year between 2005 and 2015 in Beijing, while the PM_2.5_ concentration showed a decreasing trend. The increased solar radiation, especially ultraviolet radiation, due to decreasing PM_2.5_ concentrations likely explains the increased ozone concentrations to some extent. It is also worth noting that the anthropogenic VOC (CO as proxy) decreases have been larger than those of NO_2_ over most regions of eastern China. Changes in the VOCs/NO_x_ ratio and its spatial variability may provide useful insights into the ozone-formation mechanisms over different regions [2]. A study found that the increment of summertime surface ozone was caused by decreased uptake of HO_2_ in the aerosol phase using the GEOS-Chem model in China [29]. Also, a very recent model study indicated that both VOCs and NO_x_ are important for rural ozone formation during August of 2013 in North China [30]. Considering the complexity of photochemical control, we suggest that detailed chemical and physical processes leading to increased surface-ozone concentration on a regional scale warrant further investigation both from model simulations and field observations.

**Figure 5.**
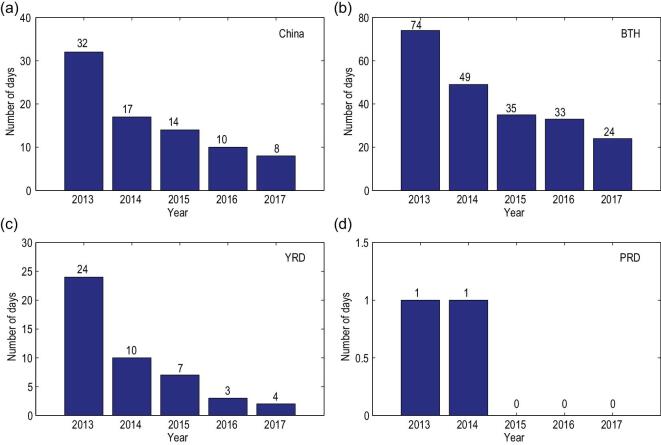
Annual variation of heavily polluted days (HPDs) over (a) China, (b) BTH, (c) YRD and (d) PRD regions. The PM_2.5_ concentrations at 74 main cities were used in the statistics. A heavily polluted day is defined as a daily averaged PM_2.5_ concentration >150 μg m^−3^ in the region.

In order to get insight into how PM_2.5_ concentration reductions affected the frequency of air-pollution episodes, we finally investigated the temporal evolution of heavily polluted days (HPDs), defined as daily PM_2.5_ mass concentration >150 μg m^−3^ (see Fig. 5). HPDs were rather frequent in 2013, with an average of 32 days over China. In the BTH region, nearly 20% of total days (74 days) were heavily polluted in 2013, followed by the YRD region (32 days). The PRD region had the most days with daily PM_2.5_ concentration <150 μg m^−3^, with only 1 HPD. The number of HPDs decreased significantly from 2013 to 2017, having values of 8, 24, 4 and 0 in China,

BTH, YRD and PRD, respectively, in 2017. We assign a significant fraction of the reduced HPD to emission reductions, even though variations in the meteorology parameters may also have contributed to the observed trend, as reported earlier for Beijing [31].

**Figure 6.**
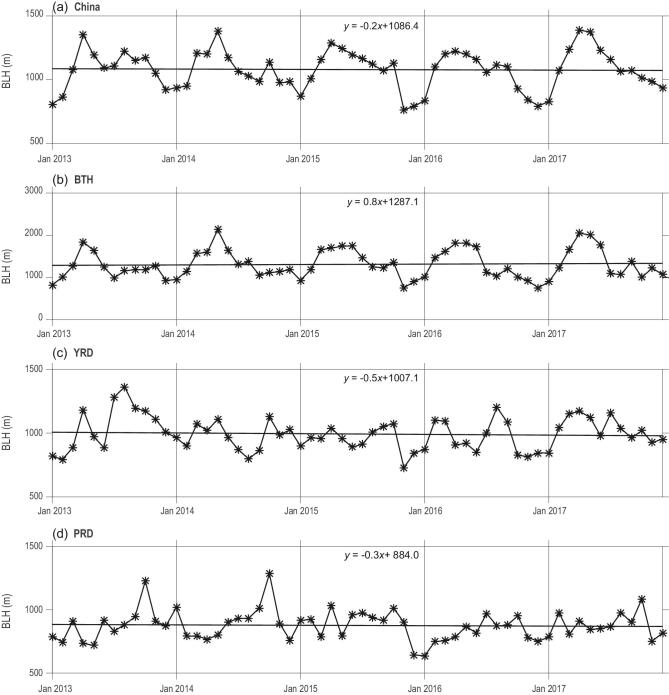
Monthly variation of BLH over (a) China, (b) BTH, (c) YRD and (d) PRD regions from 2013 to 2017.

Boundary-layer height (BLH) is a critical parameter that influences the concentrations of air pollutants [32]. To further investigate the impact of meteorology conditions on PM_2.5_ and ozone, we studied the variation of BLH from ERA-interim data from 2013 to 2017. As shown in Fig. 6, the monthly variation in BLH slightly decreased over China, YRD and PRD, while an increased trend occurred in the BTH region. However, the trends were not significant, as shown in Fig. 6. The highest increases in BLH over the BTH region since 2013 were, to some extent, due to the reduction in PM, which favored the development of BLH via increased solar radiation reaching the surface. For the other regions, the variation in BLH may be influenced by meteorology parameters and air-pollution and urban-heat island [33]. In a word, the variation in BLH from 2013 to 2017 was not the driving factor that led to decreased PM_2.5_ concentrations in eastern China, since BLH did not show significant variations as PM_2.5_ and surface ozone.

The comprehensive evaluation of PM_2.5_ mass concentrations and O_3_-mixing ratios in China from 2013 to 2017 clearly shows that the PM_2.5_ concentration significantly decreased in eastern China from 72 to 47 μg m^−3^, while the maximum average O_3_ daily concentration at the 90th percentile showed increasing trends, with the mixing ratio changing from 64 to 79 ppb. In addition, the concentrations of NO_2_, SO_2_ and CO decreased. Field-measurement data in urban Beijing showed decreased concentrations of organic aerosol, nitrate and sulfate, and that the decreased aerosol components were closely related to changes in their precursor gas concentrations. Especially in the BTH region, the concentration decreases in these components clearly suggests that the clean-air action starting from 2013 has decreased atmospheric PM pollution significantly due to strict emission controls. However, the level of photochemical pollution, measured in terms of

the ozone concentration, has gradually increased in urban areas. Our results suggest that more research and attention should be put on photochemical pollution, as well as on PM pollution, in the future.

## METHODS AND MATERIALS

The data were obtained from the Chinese National Environmental Monitoring Center (http://113.108.142.147:20035/emcpublish). The PM_2.5_ mass concentrations were measured using TEOM RP1405 (Thermo Scientific, http://www.thermoscientific.com). The resolution and precision of the instrument for 1 hour were 0.1 and ±1.5 μg m^−3^, respectively. The filters were exchanged and the flow ratio was monitored and calibrated routinely. The volume mixing ratios of ozone, SO_2_, NO_2_ and CO were measured using 49i, 43i, 42i and 48i (Thermal Environment Instruments (TEI) Inc.), respectively. The mixing ratio of the gas pollutants was calculated under standard conditions. A high-resolution time-of-flight aerosol mass spectrometer was deployed in urban Beijing to measure the chemical compositions of non-refractory submicron aerosol [34]. The campaign periods during each year are listed in the Supplementary Data file. The mass concentrations of organic, nitrate, sulfate, ammonium and chloride were recorded and averaged over 1 hour for further analysis. The ERA-interim reanalyses are assimilated results including model product and various measurements [35]. Its model-layers data contain 60 vertical layers (starting about 25 m from the surface, decreasing to about 500 m around 500 hPa), which has been used to calculate boundary-layer heights [36]. The reanalysis data with a horizontal resolution of 0.75°×0.75° and a time resolution of 6 hours were used for BLH calculation and then we interpolated the BLHs to 26 city sites.

## Supplementary Material

nwaa032_Supplemental_Files
